# Distinct scars: unique effects of physical and sexual abuse on mental health outcomes in a gender-specific substance use disorder sample in Brazil from 1998 to 2024

**DOI:** 10.1007/s00737-026-01702-5

**Published:** 2026-04-30

**Authors:** Mário Nicolau Silva Gomes, Talita Di Santi, Luisa Wolff, Sabrina Lopes Barbosa, José Vitor Tomazela, Anna Carolina Berkenbrock Mendes, Pedro Tótolo, Paulo Jeng Chian Suen, Andreza Aparecida Miranda Santos, Silvia Brasiliano, Patrícia Brunfentrinker Hochgraf, Pedro Starzynski Bacchi

**Affiliations:** 1 Institute of Psychiatry, Hospital das Clínicas da Faculdade de Medicina da Universidade de São Paulo, São Paulo, Brazil; 2 Pontifical Catholic University of Rio de Janeiro, Rio de Janeiro, Brazil; 3 Statistics Departament, Institute of Mathematics, Universidade de São Paulo, São Paulo, Brazil; 4 Laboratory of Psychiatric Neuroimaging (LIM 21), Departament of Psychiatry, Hospital das Clínicas da Faculdade de Medicina da Universidade de São Paulo, São Paulo, Brazil

**Keywords:** Women’s mental health, Substance use disorder, Physical abuse, Sexual abuse, Violence against women and girls, Gender-based violence, Gender-sensitive care, Brazil

## Abstract

**Objectives:**

(1) To compare sociodemographic characteristics, psychopathology, and substance use patterns among women diagnosed with substance use disorder (SUD) who experienced physical abuse (PA), sexual abuse (SA), or both (PSA); (2) to describe ages of onset; (3) to evaluate the impact of PA, SA, and their interaction on clinical outcomes.

**Methods:**

Observational retrospective study with patients from PROMUD (Drug Dependent Women Treatment Center) in Brazil. Questionnaires at admission assessed sociodemographics, history of SA and PA, substance use, and psychopathology. t-tests and chi-squared tests were used for descriptive statistics. Logistic and linear regressions estimated associations between clinical outcomes and history of SA or PA in crude and adjusted models. The PA–SA interaction was tested.

**Results:**

401 patients were included (1999–2024). Both SA and PA were associated with greater socioeconomic vulnerability and higher non-heterosexual orientation. SA often began in early childhood, PA more often in adolescence. PA was associated with higher odds of cocaine/crack as the main substance of dependence (OR = 2.7, *p* < 0.001) and lifetime physical aggression (OR = 1.9, *p* = 0.018). SA was linked to lifetime suicidal ideation (OR = 2.8, *p* < 0.01) and use of other substances (OR = 2.0, *p* = 0.041). No significant PA*SA interaction was found.

**Conclusions:**

SA was associated with internalizing patterns, such as suicidal ideation and cannabis/sedative use, whereas PA was linked to externalizing patterns, including physical aggression and cocaine/crack use. These effects were independent, underscoring the need to assess both in women with SUD and to develop gender-sensitive, trauma-informed interventions, particularly where resources are limited.

**Supplementary Information:**

The online version contains supplementary material available at 10.1007/s00737-026-01702-5.

## Highlights

- Women with history of abuse faced greater social and economic challenges.
- Sexual abuse independently linked to suicidal thoughts and cannabis or sedative dependence.
- Physical abuse independently linked to aggression and cocaine/crack dependence.
- Abuse often happened before age 10, with long-term impacts.
- Findings support gender-sensitive, trauma-informed addiction care.

**Supplementary Information:**

The online version contains supplementary material available at 10.1007/s00737-026-01702-5.

## Introduction

Violence Against Women and Girls (VAWG) is an overarching legal–public health category, that encompasses multiple acts, threats and coercive patterns that result in suffering to women (Falb et al. 2025). VAWG can be defined as any act of gender-based violence that results in, or is likely to result in, physical, sexual or psychological harm or suffering to women, including threats of such acts, coercion or arbitrary deprivation of liberty (Ellsberg et al. 2015). In research and clinical settings, such events are commonly assessed through histories of interpersonal abuse, which refer to patterned behaviors within relationships, such as physical abuse (PA), sexual abuse (SA), or psychological abuse (Watts and Zimmerman 2002).

Physical abuse (PA) and sexual abuse (SA) are major determinants of health, contributing substantially to disease burden across the life course (Spencer et al. 2023). Childhood, adolescence and early adulthood prove to be critical periods of heightened vulnerability. Adverse childhood experiences (ACEs), which include abuse and neglect occurring primarily before the age of 18, are important risk factors for mental health disorders (Daníelsdóttir et al. 2024). ACEs disrupt brain development involved in emotional regulation, stress response and executive function, especially during critical periods (Nelson et al. 2025). While traditional ACE frameworks emphasize a limited range of childhood adversities, expanded models also consider socioeconomic vulnerability, exposure to family violence, and household substance use exposure (Finkelhor et al. 2013). ACEs also have an impact on risk on the development of substance use disorders (SUDs), particularly in women (Martin et al. 2023), and females with SUDs report higher rates of ACE, such as PA, SA and neglect (Fletcher 2021; Carr et al. 2023).

There is evidence that women who have experienced childhood SA alone, compared to PA alone, have higher odds of alcohol dependence, and both are individually associated with increased risk of SUDs (Simpson and Miller 2002; Anne Lown et al. 2011). Moreover, studies show that having experienced both PA and SA has a cumulative effect on the risk of SUDs, and is associated with a greater addiction severity, which initiates at an earlier age, and escalates faster (Charak et al. 2015; Lotzin et al. 2019). Exposure to abuse during adulthood is also a significant risk factor, particularly among women, and longitudinal evidence suggests it independently predicts development of SUDs, even after controlling for antecedent substance disorders (Ahmadabadi et al. 2019).

Data regarding VAWG is most likely underestimated and has significant variation by region and context due to a myriad of reasons, including historical and cultural norms which may compromise data collection practices, underreporting due to stigma and fear, and methodological challenges (Palermo et al. 2014; Vahedi et al. 2023; Schredl et al. 2025). Low- and middle-income countries (LMICs) generally have higher prevalence of VAWG (Coll et al. 2020). These regions often face economic insecurity, gender inequitable norms, high levels of societal stigma, discriminatory family laws, and inadequate support services (Heise and Fulu 2015). In Brazil, a systematic review found that 20% of Brazilian women suffered physical violence in their lifetime, and one tenth in the previous year (Nakamura et al. 2023).

Nevertheless, despite evidence linking PA and SA to SUDs, the literature still lacks findings regarding the unique and independent contribution of each exposure to SUD risk and clinical presentation, particularly in clinical samples, independent of the timing of abuse across the life course. Due to the frequent co-occurrence of PA and SA, most studies either aggregate exposures or fail to estimate mutually adjusted effects, limiting inference about whether PA and SA have distinct, independent effects. Although community-based evidence suggests unique effects of PA and SA on psychopathology (Adams et al. 2018), comparable analyses in clinical samples with confirmed SUDs, particularly those adopting a gendered lens, remain scarce. Clinically relevant outcomes. like suicidality and aggressivity. are of particular interest in this context. Moreover, this evidence gap is particularly pronounced in LMICs, where women’s addiction services are often under-resourced and long-running clinical cohorts are less frequently studied.

Therefore, this study seeks to further elucidate the interplay of VAWG and SUD by analysing data from a sample of women receiving treatment for SUD in Brazil. The aims were threefold:

1. To compare sociodemographic characteristics, psychopathology, and substance use patterns among women who experienced PA, SA or both;
2. To identify the age of onset of SA and PA and examine whether the age distributions differ;
3. To evaluate the unique effect of PA, SA and their interaction on substance choice and clinical outcomes.

## Methods

### Study setting and design & sample

This observational study was conducted with patients admitted to the Drug Dependent Women Treatment Center (PROMUD) at the Institute of Psychiatry of the Hospital das Clínicas, Faculty of Medicine, University of São Paulo (IPqHCFMUSP), between 1996 and 2024. Treatment at PROMUD follows a standardized protocol which includes psychiatric care, nutritional consultations, and group psychotherapy for all patients (Leal et al. 2024; Bacchi et al. 2025). Patients were recruited if they met the inclusion and exclusion criteria of the service through a structured interview at the time of admission.

Participants were recruited from patients who voluntarily sought treatment at PROMUD. The inclusion criteria for the study were: (1) a confirmed diagnosis of SUD by a psychiatrist, according to DSM-IV or DSM-5 criteria, as applicable at the time of assessment; (2) completion of a comprehensive questionnaire administered at enrollment; and (3) informed consent obtained from the patient to participate in the study. The exclusion criterion was the absence of any responses to the specific questions regarding the history of SA or PA. All of the patients treated at PROMUD have a confirmed diagnosis of SUD by a licensed psychiatrist. No patient admitted to the service openly refused to participate in the study, but participants often missed assessments.

### Variables

Data collection was carried out through questionnaires administered at the time of admission, carried out by trained mental health professionals ensuring standardization and accuracy of the information. After the initial psychiatric interview, the main substance of dependence was determined, and posteriorly categorized into Alcohol, Cocaine/Crack, Other. The “Other” category was collapsed due to the small sample size of the individual categories, and is composed of the substances: cannabis, amphetamines, sedatives, ketamine, opioids and LSD. The questionnaire comprises sociodemographic, clinical, psychiatric and substance related questions. The semi-structured interview ASI-5 was applied from 1998 to 2005 and ASI-6 from 2006 to 2024.

The sociodemographic variables collected included age, race/ethnicity (categorized as white, black, yellow, and indigenous), education level (categorized incomplete primary school, completed primary school, completed middle school, and completed university education), occupational status (categorized as retired, employed or unemployed), marital status (partnered or not partnered), sexual orientation (heterosexual, homosexual, or bisexual and others), and financial self-sufficiency and income level (categorized as monthly income greater than one minimum wage or less than one minimum wage). Participants were assessed whether they had ever experienced suicidal thoughts or attempts, as well as any history of hetero-aggression or self-harm.

Information regarding SA was obtained through the following questions: “Did you experience any type of sexual abuse during childhood or adolescence?” and the ASI question “Have you ever been sexually assaulted/abused by someone you knew?“, while information regarding PA was obtained through the following question: “Have you ever been physically assaulted/abused by someone you knew?“. Patients who reported having experienced SA at any of these questions were allocated to the “SA” group, patients who reported having experienced PA at any of these questions were allocated to the “PA” group, patients who experienced both types of abuse were allocated to the “SA and PA” group, while those who denied any history of abuse were allocated to the “No abuse” group. The ages of onset of these abuses were assessed by the question “How old were you when this happened for the first time?”, following both the questions of SA and PA.

### Statistical analyses

Analyses were performed using R software version 4.2. For our first aim, The four groups (No Abuse / SA / PA / SA and PA) were compared. To compare the means of continuous variables across the groups, we performed an ANOVA. For categorical variables, a Chi-square test (χ²) was used. The level of statistical significance was set at *p* < 0.05. For our second aim Kernel Density Estimates for the age distribution at onset of SA and PA were plotted and a Kolmogorov-Smirnov test was used to evaluate differences between distributions.

For our third aim, linear regression betas and 95% confidence intervals (95% CI) were estimated for the associations between history of sexual abuse (SA) or physical abuse (PA) and the age at which the patient entered PROMUD. Logistic regressions were used to estimate odds ratios and 95% confidence intervals for the associations between a history of SA or PA and various clinical outcomes. We first built a crude model for each outcome, including only SA and PA as predictors. Subsequently, we built an adjusted model, including sex, educational level, and year of admission as covariates. Year of admission was included as a covariate to account for secular trends over the cohort’s three-decade span, including changes in diagnostic frameworks for substance use disorders (DSM-IV to DSM-5, shifts in violence reporting practices (Lima et al. 2025) and greater public and clinical awareness (Palermo et al. 2014), and changes in drug-use patterns over time (Malta et al. 2021).

All models were also tested for an interaction between SA and PA; The models with interaction had the following formula: Outcome ~ PA * SA + confounders. However, these interactions were not statistically significant and thus not included in the final reported models. A detailed breakdown of the interaction terms and their p-values can be found in Table S2 and S3.

Item-level missingness in sociodemographic and clinical variables was assumed to be Missing at Random (MAR), as missingness was plausibly related to observed factors such as year of admission, educational level, and interview version, all of which were accounted for.

Multicollinearity was assessed using Variance Inflation Factors (VIF), with all values below 3 indicating stable standard errors in the models with no interactions. When interaction terms are added, VIF values are naturally much higher. Dispersion and outlier tests were conducted using simulation-based diagnostics via the DHARMa package, suggesting no assumption violations **(Tables ****S4**** and ****S5****).**

### Ethics statement

The study was approved by the Research Ethics Committee of the Institute of Psychiatry of the Hospital das Clínicas, Faculty of Medicine, University of São Paulo (IPqHCFMUSP). All participants signed the Informed Consent Form (ICF) prior to inclusion in the study. The authors assert that all procedures in this work comply with the ethical standards of the relevant national and institutional committees on human experimentation and with the Helsinki Declaration of 1975, as revised in 2008.

## Results

From the initial sample of 854 patients, 200 were excluded because the ASI questionnaire was applied only from 1998 on, and 253 were excluded due to missing data on PA or SA. Therefore, 401 patients were included, according to history of abuse: no abuse (NA, *n* = 135; 33.6%), physical abuse (PA, *n* = 92; 22.9%), sexual abuse (SA, *n* = 71; 17.7%), and both physical and sexual abuse (PSA, *n* = 103; 25.6%). Mean age at admission differed significantly between groups (*p* = 0.026), being highest in the NA group. Additionally, the year of admission to PROMUD differed significantly (*p* = 0.002), with a predominance of NA between 1998 and 2006 (46.7%) and a predominance of patients with a history of some type of abuse between 2014 and 2024 (ranging from 23.9% to 45.6%). A significant difference in sexual orientation (*p* = 0.012) was observed, with higher rates of bisexuality in abuse groups (9.6%–21.2%) compared to NA (4.2%), and lower prevalence of heterosexuality in the abuse groups (Table 1). Self-reported income insufficiency was also significantly associated with abuse history (*p* = 0.002), reported by 64.7% to 74.2% in abuse groups compared to 45.8% in the NA group, indicating higher socioeconomic vulnerability in those with abuse experiences. Additionally, a significant association was observed between monthly income and a history of abuse (*p* = 0.018). Among patients with reported abuse, 33.3% to 51.4% reported receiving more than one minimum wage per month, whereas 64.5% of patients without a history of abuse reported earning more than one minimum wage per month. (Table 1).

No significant differences were found regarding race, marital status, education, employment, income, age at alcohol use onset, prior treatment, or secondary alcohol dependence. However, a significant difference was found for the primary substance of dependence (*p* < 0.001), with cocaine/crack more prevalent in PA (39.1%), SA (24.3%), and PSA (39%) than in NA (15%). Lifetime suicidal ideation was significantly more frequent (*p* < 0.001) in PA (56.2%), SA (70.6%), and PSA (70.7%) compared to NA (37.2%). Reports of lifetime physical aggression were also significantly higher in abuse groups (*p* = 0.024), especially in PSA (51.8%) versus NA (28.9%) (Table 1).

**Table 1:** Characteristics of the sample

Characteristic	No abuse (*N* = 135)	Physical abuse (*N* = 92)	Sexual abuse (*N* = 71)	Sexual and physical abuse (*N* = 103)	*P*-value	Missing (%)
Age at entry (mean ± SD)	42.79 ± 11.99	39.22 ± 12.31	38.79 ± 11.75	38.71 ± 12.04	0.026	0%
Year of admission to PROMUD					0.002	
From 1998 to 2006	63 (46.7%)	34 (37%)	26 (37.1%)	27 (26.2%)		
From 2006 to 2014	38 (28.1%)	36 (39.1%)	16 (22.9%)	29 (28.2%)		
From 2014 to 2024	34 (25.2%)	22 (23.9%)	28 (40%)	47 (45.6%)		
Race (%)					0.329	1%
Yellow	3 (2.3%)	0 (0.0%)	2 (2.8%)	1 (1.0%)		
White	101 (75.9%)	61 (67.0%)	54 (76.1%)	71 (69.6%)		
Indigenous	0 (0.0%)	1 (1.1%)	0 (0.0%)	0 (0.0%)		
Black	29 (21.8%)	29 (31.9%)	15 (21.1%)	30 (29.4%)		
Answered “no partner” for marital status (%)	90 (67.7%)	65 (70.7%)	45 (69.2%)	82 (80.4%)	0.163	2.2%
Sexual Orientation (%)					0.012	13.2%
Bisexual and others	5 (4.2%)	7 (9.6%)	14 (21.2%)	11 (12.1%)		
Heterosexual	106 (89.8%)	57 (78.1%)	46 (69.7%)	71 (78.0%)		
Homosexual	7 (5.9%)	9 (12.3%)	6 (9.1%)	9 (9.9%)		
Financial self-sufficiency (%)	45 (54.2%)	16 (25.8%)	12 (35.3%)	26 (31.0%)	0.002	34.4%
Income greater minimum wage (%)	49 (64.5%)	29 (46.8%)	11 (33.3%)	38 (51.4%)	0.018	38.9%
Occupational status (%)					0.398	8.5%
Retired	8 (6.6%)	5 (6.1%)	8 (11.6%)	6 (6.3%)		
Unemployed	57 (47.1%)	45 (54.9%)	38 (55.1%)	43 (45.3%)		
Employed	56 (46.3%)	32 (39.0%)	23 (33.3%)	46 (48.4%)		
Education Level (%)					0.467	4.7%
Incomplete primary	16 (12.2%)	10 (11.1%)	10 (15.4%)	15 (15.6%)		
Complete primary	14 (10.7%)	12 (13.3%)	14 (21.5%)	14 (14.6%)		
Complete primary	62 (47.3%)	46 (51.1%)	31 (47.7%)	44 (45.8%)		
Complete university education	39 (29.8%)	22 (24.4%)	10 (15.4%)	23 (24.0%)		
Main substance (%)					< 0.001	2.7%
Alcohol	95 (71.4%)	45 (51.7%)	40 (57.1%)	44 (44.0%)		
Other	18 (13.5%)	8 (9.2%)	13 (18.6%)	17 (17.0%)		
Cocaine and crack	20 (15.0%)	34 (39.1%)	17 (24.3%)	39 (39.0%)		
Age of onset of alcohol use (mean ± SD)	19.96 ± 10.18	18.63 ± 8.07	17.71 ± 8.67	16.96 ± 7.72	0.091	11.2%
Previous treatment for alcohol (%)	40 (50.6%)	24 (48.0%)	20 (50.0%)	27 (41.5%)	0.719	41.6%
Previous treatment for other substance (%)	31 (40.8%)	24 (47.1%)	26 (54.2%)	39 (54.2%)	0.335	38.4%
SADD Score (mean ± SD)	18.77 ± 11.86	17.37 ± 10.91	19.42 ± 12.41	18.99 ± 12.75	0.814	30.7%
Lifetime suicidal ideation (%)	32 (37.2%)	36 (56.2%)	24 (70.6%)	58 (70.7%)	< 0.001	33.7%
Lifetime suicide attempt (%)	21 (32.8%)	20 (44.4%)	22 (51.2%)	33 (50.0%)	0.164	45.6%
Lifetime physical aggression (%)	24 (28.9%)	28 (43.8%)	13 (37.1%)	43 (51.8%)	0.024	33.9%

Table of sociodemographic and behavioral data, in which ANOVA was used for continuous variables and the chi-square test was applied for categorical variables

**Fig. 1:**
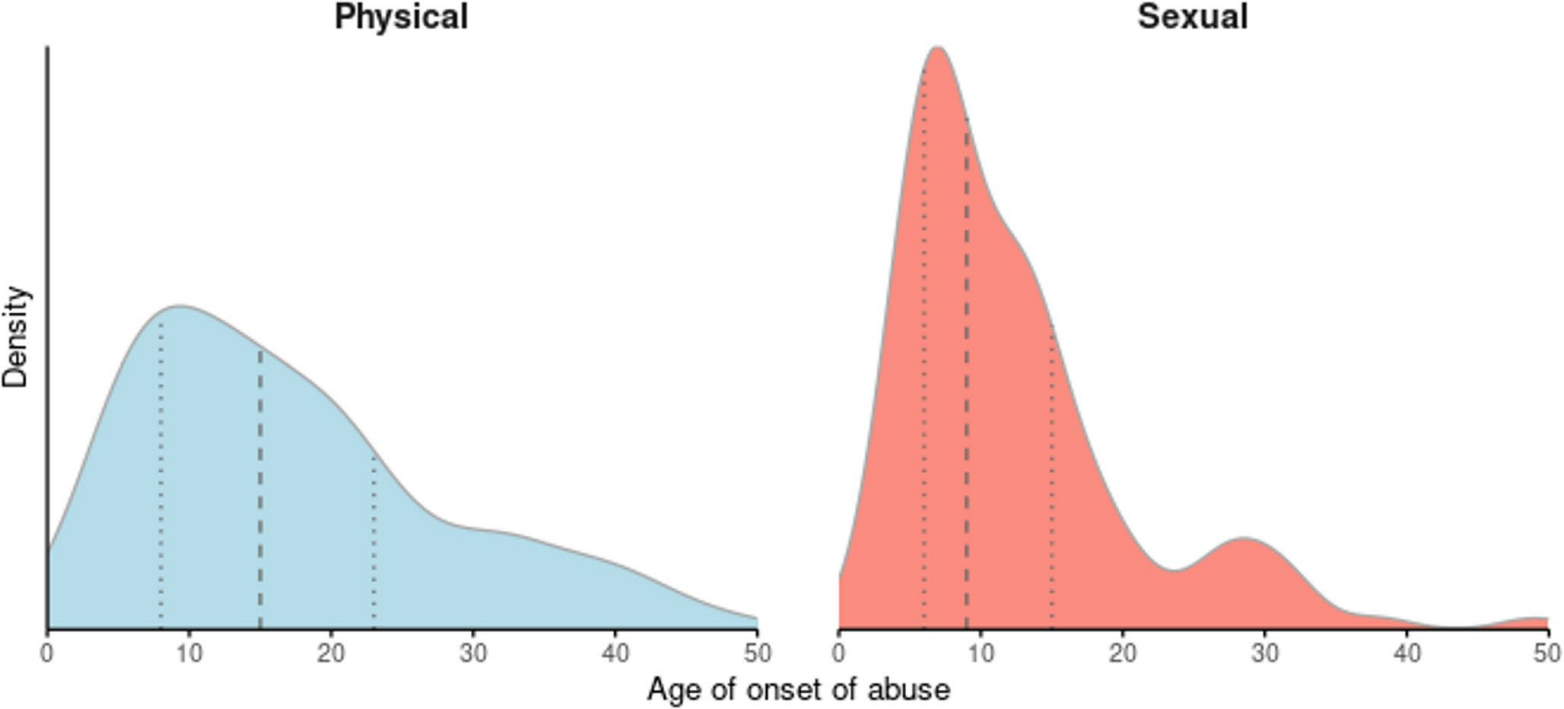
Kernel Density Estimates showing the age distribution at onset of sexual and physical violence. Dashed lines indicate quartiles. A Kolmogorov-Smirnov test rejected the null hypothesis of equality of these two distributions (p < 0.001), indicating a statistically significant difference in the age profiles of sexual versus physical violence onset

Kernel Density Estimates showing the age distribution at onset of sexual and physical violence. Dashed lines indicate quartiles. A Kolmogorov-Smirnov test rejected the null hypothesis of equality of these two distributions (*p* < 0.001), indicating a statistically significant difference in the age profiles of sexual versus physical violence onset.

**Fig. 2:**
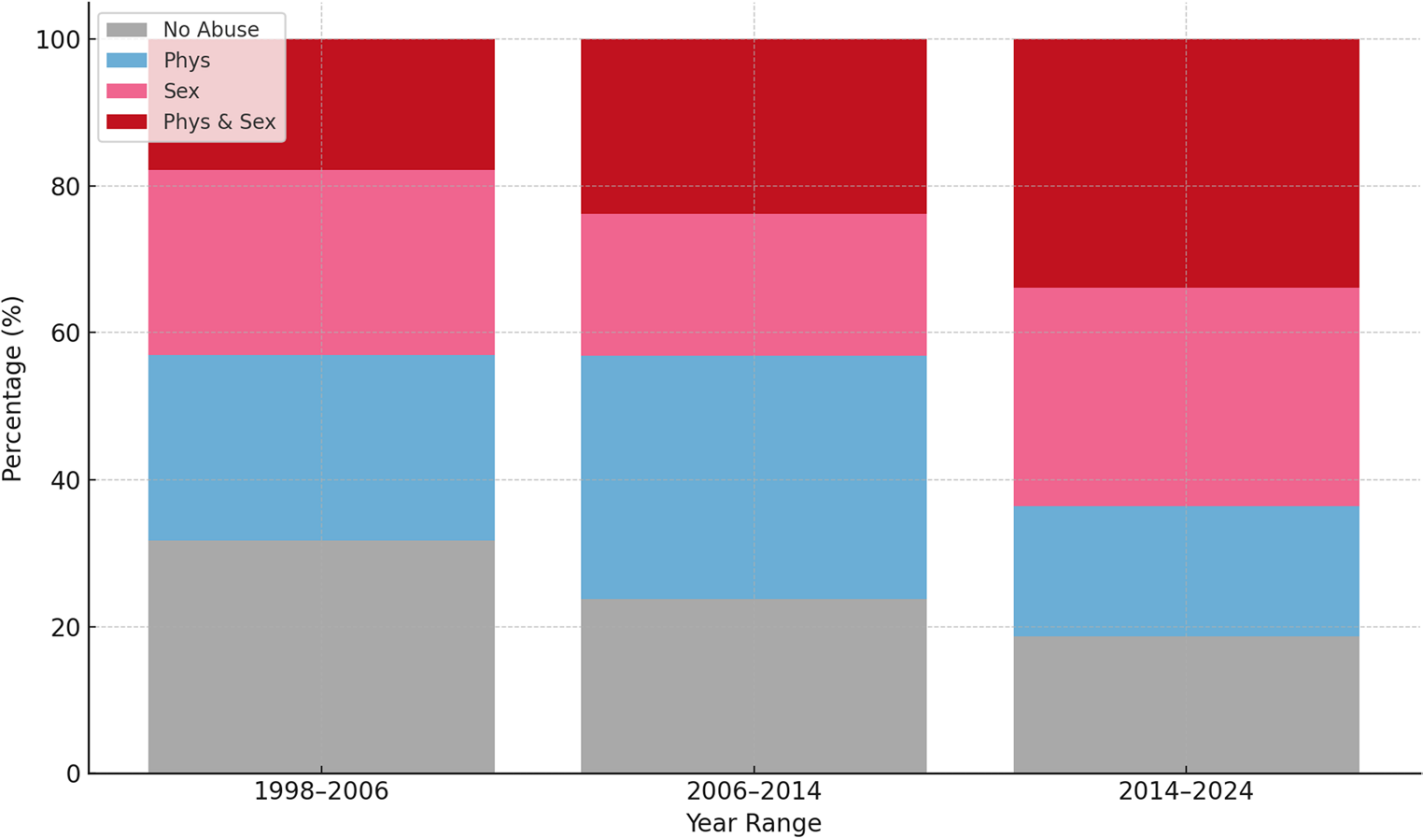
Sample distribution of the number of abuse reports by patient arrival treatment period, stratified by abuse type, with a notable increase in simultaneous sexual and physical abuse over time

Sample distribution of the number of abuse reports by patient arrival treatment period, stratified by abuse type, with a notable increase in simultaneous sexual and physical abuse over time.

**Table 2:** Estimates for interaction effect between sexual and physical abuse on outcome

Outcome variable	Model scenario	Physical: sexual abuse interaction estimate	*P*-value
Age at Admission	Crude	3.5	0.15
Age at Admission	Adjusted	3.8	0.14
Lifetime suicide attempt	Crude	-0.5	0.33
Lifetime suicide attempt	Adjusted	-0.6	0.27
Lifetime suicidal ideation	Crude	-0.8	0.17
Lifetime suicidal ideation	Adjusted	-0.8	0.17
Lifetime physical aggression	Crude	0.0	0.92
Lifetime physical aggression	Adjusted	0.2	0.73
Main substance - other	Crude	0.2	0.70
Main substance - cocaine/crack	Crude	-0.5	0.27
Main substance - other	Adjusted	0.4	0.58
Main substance - cocaine/crack	Adjusted	-0.8	0.12

Estimates for interaction of sexual and physical abuse as predictors for multiple outcomes, and p values showing that none was significant

Estimates for interaction of sexual and physical abuse as predictors for multiple outcomes, and p values showing that none was significant.

**Fig. 3:**
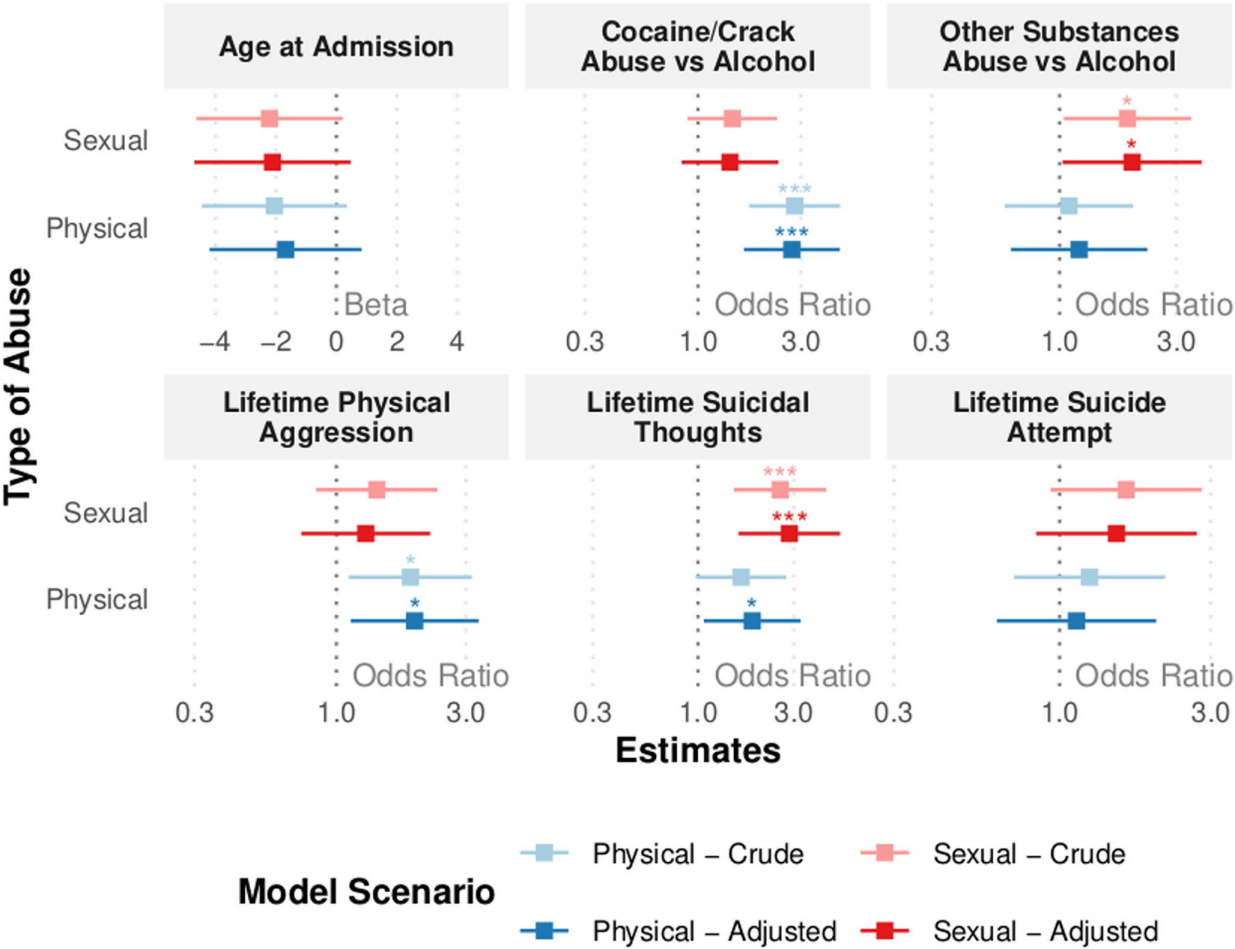
Forest Plot of the effect of sexual abuse or physical on different outcomes. First, crude effects were established, afterwards, a model adjusted by race, education level and year of treatment. This model did not consider interaction terms between sexual abuse and physical abuse

Forest Plot of the effect of sexual abuse or physical on different outcomes. First, crude effects were established, afterwards, a model adjusted by race, education level and year of treatment. This model did not consider interaction terms between sexual abuse and physical abuse.

For the regression analyses, the first model tested the interaction between SA and PA; no significant interaction term was found for any outcome (Table 2; complete results in **Table ****S1****)**. We then proceeded to the model without interaction terms. Regarding the outcome “age at admission”, no significant effect was found. For the outcome “use of cocaine/crack relative to alcohol as the primary substance of abuse,” a significant association was observed with PA (*p* < 0.001, adjusted OR = 2.7). SA was significantly associated with Other Substances ( *p* = 0.041, adjusted OR = 2.0 ) (Fig. 3; complete results in **Table ****S2**).

PA was associated with higher odds of “lifetime history of physical aggression,” (adjusted OR = 1.9). Regarding suicidal ideation, SA showed a strong positive association (adjusted OR = 2.8). PA was also significantly associated in the adjusted model (*p* = 0.028), although with a lower magnitude (OR = 1.9). Finally, for the outcome “lifetime suicide attempt”, no significant effect was found.

Compared with non-responders, responders were older at entry (40.21 ± 12.13 vs. 37.49 ± 12.54 years; *p* = 0.002) and differed by admission period (*p* = 0.002), with higher income above the minimum wage (51.8% vs. 36.7%; *p* = 0.012), employment (51.8% vs. 33.0%; *p* = 0.003), and education (*p* < 0.001), while race (*p* = 0.796), marital status (*p* = 0.163), main substance (*p* = 0.067), and clinical variables (all *p* > 0.05) were similar. These results can be seen in the **Table ****S3**.

## Discussion

For our first aim we compared sociodemographic characteristics, psychopathology, and substance use patterns among women who experienced PA, SA or both. Two thirds of the sample had suffered at least one type of abuse. Women exposed to abusive experiences, particularly those in the PSA group, have higher socioeconomic vulnerability, including being unemployed and self-declaring financially insufficient. These findings are consistent with robust evidence that VAWG poses higher risk for adverse economic outcomes (Sauber and O’Brien 2020; Domond et al. 2023). We found that women with a history of at least one type of abuse were more likely to identify as non-heterosexual. The role of sexual orientation and its relationship with abuse is a complex issue, and there seem to be higher rates of child abuse among non-heterosexual individuals (Koeppel and Bouffard 2014). Women enrolled in the program between 2013 and 2024 presented higher abuse rates, especially SA. There is evidence of increased SA reporting in Brazil in the last decades, which likely reflects both greater awareness and improved reporting mechanisms, rather than a true rise in incidence (Gaspar and Pereira 2018).

Our second aim was to analyze and compare the age of onset of SA and PA. Half of the SAs occurred firstly before 10 years, and half of PAs occurred firstly before 15 years. Such findings are in line with clinical and community samples in LMIC, where first SA typically occurred between 6 and 12 years old (Rueda et al. 2021). In the context of SUDs, childhood abuses are associated with greater severity, earlier initiation of drug use and psychiatric comorbidities (Valério et al. 2022; Rakovski et al. 2024). Among women with SUD, physical abuse frequently begins during early developmental periods and is strongly associated with earlier initiation of substance use and greater clinical severity, especially when accompanied by comorbid PTSD (Lansford et al. 2010; Lotzin et al. 2019).

Adverse Childhood Events (ACEs), including PA and SA, impact the developing brain and social cognition (O’Hare et al. 2023) and are implicated in genetic, epigenetic, cultural, and psychosocial mechanisms (Donovan et al. 2024). When addressing substance use, the impact becomes increasingly complex and bidirectional. Exposure to traumatic events can induce stress, fear and isolation and, on the other hand, women with severe mental health issues are at a higher risk of experiencing violent victimization (Hyde et al. 2008; Khalifeh and Dean 2010).

For our third aim, we evaluated the impact of PA, SA, and their interaction on clinical and behavioral outcomes. Women who suffered SA were approximately three times as likely to have had suicidal ideation during their lifetime, suggesting a significantly high predisposition to psychological suffering and suicidal thoughts. Patients with a history of PA were also linked to higher rates of suicidal ideation, although with a less pronounced effect.

ACEs present both direct and indirect effects on suicidality (Kim et al. 2021). Epidemiological studies and meta-analyses demonstrate an association between childhood SA and a 2.5–3-fold increase in the odds of suicidal ideation and attempts in both youth and adults (Angelakis et al. 2020; Dworkin et al. 2022). There is also evidence of a smaller, 2-fold increased risk for these outcomes, among those who suffered PA (Visioli et al. 2023). In women, suicidal thoughts may originate from stigma and neglect, whether in childhood or later, during sociocultural integration. Social determinants may perpetuate suicidality among vulnerable populations (Na et al. 2025). Sexually abused women have elevated internalizing symptoms, such as depression and PTSD (Yang et al. 2023; Hamel et al. 2024), which could mediate relationships between ACEs and later risk behaviours, such as SUD (Villar et al. 2024).

PA was associated with an increased chance of physical aggression during lifetime. Victims of PA may present violent behaviours during their lifetime (Stouthamer-Loeber et al. 2001), especially during adolescence (Smith and Thornberry 1995). The sentence “Violence begets violence” has been studied since the end of the last century (Widom 1989). Children who have been physically abused present higher risks of continuity of violent behaviours towards their own children later in life (Freisthler et al. 2017). PA is generally associated with externalizing symptoms and behaviours (Aksoy et al. 2022; Hamel et al. 2024). The use of cocaine is also associated with externalizing traits (Vorspan et al. 2020), and individuals who use cocaine have an increased chance of both suffering and perpetrating PA (Lee et al. 1997; Kadri et al. 2025). Our findings support this complex dynamic, once women in our sample that suffered PA are more prone to be mainly cocaine users.

We did not find interaction between PA and SA as predictors, which most likely means they act independently, in a cumulative but not interacting manner, neither attenuating nor exacerbating each other as exposures. We hereby posit that these two direct risk factors should be assessed and independently accounted for in clinical settings.

### Strengths

To our knowledge, this was the first study to examine unique effects of physical and sexual as predictors of psychopathology in a clinical sample of women with SUD.

This study has data from women spanning 26 years and is to our knowledge the largest of such clinical samples in Brazil. Additionally, a gender-specific center offers a unique opportunity to gain insights into a historically underrepresented population. Therefore, the findings of our study may have broader relevance and applicability to similar contexts, particularly in other LMICs.

### Limitations

The limitations of the study must be stated. PTSD prevalence and association with SUD were not accounted for in our sample, despite its role as a possible mediator between victimization and the severity of various types of SUDs. Another limitation of our study lies in the patients’ understanding of what constitutes an abusive situation. It is undeniable that throughout the analyzed period, there have been tangible societal and cultural shifts which may, in turn, influence reporting of abuse.

Furthermore, the present study relies on self-report data, which may incur recall bias, which is particularly important for reporting past traumatic events. Sociodemographic characteristics of our sample, including a predominance of higher education levels than Brazilian average, higher income, and white race/ethnicity, may hinder the external validity of our findings.

Additionally, we could not adjust for some contextual factors that may influence both exposure to violence and clinical outcomes, including social support, family support, and family history of mental health disorders.

### Conclusion

The present study provides evidence of the associations between SA, PA, and adverse clinical outcomes in women with SUD. Both SA and PA were correlated with greater socioeconomic vulnerability and frequently occurred early in life. SA was associated with internalizing patterns, such as suicidal ideation and use of substances like cannabis and sedatives, and PA was associated with externalizing patterns, like physical aggression and cocaine or crack use. The independence of the effects of PA and SA suggests that these events should be carefully assessed and interpreted in a clinical setting, to further develop gender-sensitive and trauma-informed tailored care.

## Supplementary Information

Below is the link to the electronic supplementary material.

Supplementary Material 1

Supplementary Material 2

## Data Availability

No datasets were generated or analysed during the current study.
