# Supplementary material for: Distinct scars: unique effects of physical and sexual abuse on mental health outcomes in a gender-specific substance use disorder sample in Brazil from 1998 to 2024
Source: Arch Womens Ment Health. 2026 Apr 30;29(3):70. doi: 10.1007/s00737-026-01702-5 (PMC13132932; doi:10.1007/s00737-026-01702-5)
Supplement: Supplementary file 1 — Supplementary Material 1 [file 737_2026_1702_MOESM1_ESM.docx]

**Supplementary Table 1**

| **Sample size** | **Outcome variable** | **Model type** | **Model scenario** | **Physical estimate** | **Sexual estimate** | **Phy:Sex estimate** | **Phy standard error** | **Sex standard error** | **Phy:Sex standard error** | **Physical p value** | **Sexual p value** | **Phy:Sex p value** | **OR PA conditioned on no report of SA** | **OR PA conditioned on the presence of SA reports** | **OR SA conditioned on no report of PA** | **OR SA conditioned on the presence of PA reports** |
| --- | --- | --- | --- | --- | --- | --- | --- | --- | --- | --- | --- | --- | --- | --- | --- | --- |
| 401 | Age t0 | gaussian | Interaction Only | -3.6 | -4.0 | 3.5 | 1.63 | 1.76 | 2.47 | 0.0289 | 0.0241 | 0.1586 |  |  |  |  |
| 379 | Age t0 | gaussian | Controlled | -3.3 | -4.1 | 3.8 | 1.68 | 1.88 | 2.58 | 0.0526 | 0.0309 | 0.1454 |  |  |  |  |
| 218 | Lifetime suicide attempt | binomial | Interaction Only | 0.5 | 0.8 | -0.5 | 0.40 | 0.40 | 0.56 | 0.2185 | 0.0594 | 0.3356 | 1.6 | 1.0 | 2.1 | 1.3 |
| 207 | Lifetime suicide attempt | binomial | Controlled | 0.4 | 0.8 | -0.6 | 0.41 | 0.43 | 0.59 | 0.2889 | 0.0814 | 0.2741 | 1.5 | 0.8 | 2.1 | 1.1 |
| 266 | Lifetime suicidal ideation | binomial | Interaction Only | 0.8 | 1.4 | -0.8 | 0.34 | 0.44 | 0.56 | 0.0214 | 0.0014 | 0.1706 | 2.2 | 1.0 | 4.1 | 1.9 |
| 254 | Lifetime suicidal ideation | binomial | Controlled | 0.9 | 1.5 | -0.8 | 0.36 | 0.48 | 0.60 | 0.0108 | 0.0014 | 0.1783 | 2.5 | 1.1 | 4.7 | 2.1 |
| 265 | Lifetime physical aggression | binomial | Interaction Only | 0.6 | 0.4 | 0.0 | 0.35 | 0.43 | 0.54 | 0.0636 | 0.3801 | 0.9267 | 1.9 | 1.8 | 1.5 | 1.4 |
| 253 | Lifetime physical aggression | binomial | Controlled | 0.6 | 0.1 | 0.2 | 0.36 | 0.46 | 0.57 | 0.0985 | 0.7847 | 0.7335 | 1.8 | 2.2 | 1.1 | 1.4 |
| 390 | Main substance - Others | multinomial | Interaction Only | -0.1 | 0.5 | 0.2 | 0.46 | 0.41 | 0.63 | 0.8903 | 0.1880 | 0.7071 | 0.9 | 1.2 | 1.7 | 2.2 |
| 390 | Main substance - cocaine and crack | multinomial | Interaction Only | 1.3 | 0.7 | -0.5 | 0.33 | 0.38 | 0.49 | 0.0001 | 0.0645 | 0.2721 | 3.6 | 2.1 | 2.0 | 1.2 |
| 368 | Main substance - Others | multinomial | Controlled | -0.1 | 0.5 | 0.4 | 0.49 | 0.45 | 0.67 | 0.9167 | 0.2348 | 0.5870 | 0.9 | 1.4 | 1.7 | 2.5 |
| 368 | Main substance - cocaine and crack | multinomial | Controlled | 1.4 | 0.8 | -0.8 | 0.35 | 0.41 | 0.52 | 0.0001 | 0.0397 | 0.1274 | 3.9 | 1.8 | 2.3 | 1.0 |

*Summary of regression model results for physical and sexual abuse exposure on different outcomes. Models include main effects and interaction terms (physical × sexual abuse), with unadjusted and adjusted conditional odds ratios (CORs) and corresponding standard errors and p-values. Sample size, outcome variable and model type are reported for each analysis.*

**Supplementary Table 2**

| **Sample size** | **Outcome variable** | **Model type** | **Model scenario** | **Physical estimate** | **Sexual estimate** | **Phy standard error** | **Sex standard error** | **Physical p value** | **Sexual p value** | **Physical Odds Ratio** | **Sexual Odds Ratio** |
| --- | --- | --- | --- | --- | --- | --- | --- | --- | --- | --- | --- |
| 401 | Age t0 | gaussian | Interaction Only | -2.1 | -2.2 | 1.23 | 1.24 | 0.0947 | 0.0739 |  |  |
| 379 | Age t0 | gaussian | Controlled | -1.7 | -2.1 | 1.28 | 1.32 | 0.1911 | 0.1097 |  |  |
| 218 | Lifetime suicide attempt | binomial | Interaction Only | 0.2 | 0.5 | 0.28 | 0.28 | 0.4373 | 0.0844 | 1.2 | 1.6 |
| 207 | Lifetime suicide attempt | binomial | Controlled | 0.1 | 0.4 | 0.30 | 0.30 | 0.6772 | 0.1647 | 1.1 | 1.5 |
| 266 | Lifetime suicidal ideation | binomial | Interaction Only | 0.5 | 0.9 | 0.26 | 0.27 | 0.0621 | 0.0005 | 1.6 | 2.6 |
| 254 | Lifetime suicidal ideation | binomial | Controlled | 0.6 | 1.0 | 0.28 | 0.30 | 0.0280 | 0.0004 | 1.9 | 2.8 |
| 265 | Lifetime physical aggression | binomial | Interaction Only | 0.6 | 0.3 | 0.27 | 0.26 | 0.0187 | 0.1927 | 1.9 | 1.4 |
| 253 | Lifetime physical aggression | binomial | Controlled | 0.7 | 0.2 | 0.28 | 0.28 | 0.0165 | 0.3732 | 1.9 | 1.3 |
| 390 | Main substance - cocaine and crack | multinomial | Interaction Only | 1.0 | 0.4 | 0.25 | 0.24 | 0.0000 | 0.1330 | 2.8 | 1.4 |
| 368 | Main substance - Others | multinomial | Controlled | 0.2 | 0.7 | 0.33 | 0.33 | 0.5737 | 0.0411 | 1.2 | 2.0 |
| 390 | Main substance - Others | multinomial | Interaction Only | 0.1 | 0.6 | 0.31 | 0.31 | 0.7750 | 0.0371 | 1.1 | 1.9 |
| 368 | Main substance - cocaine and crack | multinomial | Controlled | 1.0 | 0.3 | 0.26 | 0.26 | 0.0001 | 0.1949 | 2.7 | 1.4 |

*Summary of regression model results for physical and sexual abuse exposure on different outcomes. Models include main effects, excluding interaction terms following non-significant effects, with unadjusted and adjusted odds ratios (ORs) and corresponding standard errors and p-values. Sample size, outcome variable and model type are reported for each analysis.*
