# Supplementary material for: Distinct scars: unique effects of physical and sexual abuse on mental health outcomes in a gender-specific substance use disorder sample in Brazil from 1998 to 2024
Source: Arch Womens Ment Health. 2026 Apr 30;29(3):70. doi: 10.1007/s00737-026-01702-5 (PMC13132932; doi:10.1007/s00737-026-01702-5)
Supplement: Supplementary file 2 — Supplementary Material 2 [file 737_2026_1702_MOESM2_ESM.docx]

**Supplementary Material 2**

**Table S3.** Responders vs non responders

| **Characteristic** | **Non-responder**  **(N=364)** | **Responder**  **(N=401)** | **P-value** |
| --- | --- | --- | --- |
| **Age at entry (mean ± SD)** | 37.49 ± 12.54 | 40.21 ± 12.13 | 0.002 |
| **Year of admission (%)** |  |  | 0.002 |
| From 1998 to 2006 | 34 (37%) | 26 (37.1%) |  |
| From 2006 to 2014 | 36 (39.1%) | 16 (22.9%) |  |
| From 2014 to 2024 | 22 (23.9%) | 28 (40%) |  |
| **Race (%)** |  |  | 0.796 |
| Yellow | 2 (0.8%) | 6 (1.5%) |  |
| White | 194 (74.3%) | 287 (72.3%) |  |
| Indigenous | 1 (0.4%) | 1 (0.3%) |  |
| Black | 64 (24.5%) | 103 (25.9%) |  |
| **Marital Status - “No Partner” (%)** | 132 (62.9%) | 282 (71.9%) | 0.163 |
| **Sexual Orientation (%)** |  |  | 0.012 |
| Bisexual and others | 8 (5.2%) | 37 (10.6%) |  |
| Heterosexual | 136 (88.3%) | 280 (80.5%) |  |
| Homosexual | 10 (6.5%) | 31 (8.9%) |  |
| **Income higher than minimum wage (%)** | 40 (36.7%) | 127 (51.8%) | 0.012 |
| **Occupational status (%)** |  |  | 0.003 |
| Retired | 11 (5.9%) | 27 (7.4%) |  |
| Unemployed | 87 (47.0%) | 162 (44.1%) |  |
| Employed | 61 (33.0%) | 157 (51.8%) |  |
| Homemaker | 26 (14.1%) | 21 (5.7%) |  |
| **Education Level (%)** |  |  | <0.001 |
| Incomplete primary | 69 (29.9%) | 51 (13.4%) |  |
| Complete primary | 49 (21.2%) | 54 (14.1%) |  |
| Complete primary | 81 (35.1%) | 183 (47.9%) |  |
| Complete university education | 32 (13.9%) | 94 (24.6%) |  |
| **Main substance (%)** |  |  | 0.067 |
| Alcohol | 181 (51.9%) | 224 (57.4%) |  |
| Other | 42 (12.0%) | 56 (14.4%) |  |
| Cocaine and crack | 126 (36.1%) | 110 (28.2%) |  |
| **Age of onset of alcohol use (mean ± SD)** | 19.51 ± 9.84 | 18.46 ± 8.88 | 0.291 |
| **Previous treatment for alcohol (%)** | 45 (44.6) | 111 (47.4) | 0.714 |
| **Previous treatment for other substance (%)** | 31 (38.3) | 120 (48.6) | 0.137 |
| **SADD Score (mean ± SD)** | 17.90 ± 10.06 | 18.65 ± 11.98 | 0.743 |
| **Lifetime suicidal ideation (%)** | 1 (33.3) | 150 (56.4) | 0.83 |
| **Lifetime suicide attempt (%)** | 68 (45.6) | 96 (44.0) | 0.845 |
| **Lifetime physical aggression (%)** | 0 (0.0) | 108 (40.8) | 0.655 |

Table of sociodemographic and clinical data comparing responders and non responders of the main exposures (PA and SA). Student’s t-test was used for continuous variables and chi-square test was applied for categorical variables.

**Table S4.** Model diagnostics. No interaction.

| **Outcome Variable** | **Model Scenario** | **Maximum VIF** | **Dispersion Test P-value*** | **Outlier Test P-value*** |
| --- | --- | --- | --- | --- |
| Age at Admission | Crude | 1.04 | 0.89 | 0.78 |
| Age at Admission | Adjusted | 1.19 | 0.78 | 0.13 |
| Lifetime suicide attempt | Crude | 1.03 | 0.75 | 1 |
| Lifetime suicide attempt | Adjusted | 1.17 | 0.9 | 1 |
| Lifetime suicidal ideation | Crude | 1.05 | 0.98 | 1 |
| Lifetime suicidal ideation | Adjusted | 1.21 | 0.86 | 1 |
| Lifetime physical aggression | Crude | 1.06 | 0.83 | 1 |
| Lifetime physical aggression | Adjusted | 1.17 | 0.8 | 1 |
| Main substance | Crude | 2.31 | 0.82 | 1 |
| Main substance | Adjusted | 2.96 | 0.95 | 1 |

* Dispersion test and outlier test are simulation based, using randomized quantile residuals in the DHARMa package.

**Table S5.** Model diagnostics. With interaction.

| **Outcome Variable** | **Model Scenario** | **Maximum VIF** | **Dispersion Test P-value*** | **Outlier Test P-value*** |
| --- | --- | --- | --- | --- |
| Age at Admission | Crude | 3.22 | 0.85 | 0.57 |
| Age at Admission | Adjusted | 3.23 | 0.74 | 0.13 |
| Lifetime suicide attempt | Crude | 3.56 | 0.85 | 1 |
| Lifetime suicide attempt | Adjusted | 3.55 | 0.88 | 1 |
| Lifetime suicidal ideation | Crude | 3.82 | 0.9 | 1 |
| Lifetime suicidal ideation | Adjusted | 3.81 | 0.88 | 1 |
| Lifetime physical aggression | Crude | 4.03 | 0.83 | 1 |
| Lifetime physical aggression | Adjusted | 4.1 | 0.8 | 1 |
| Main substance | Crude | 5.6 | 1 | 1 |
| Main substance | Adjusted | 5.88 | 0.73 | 1 |

* Dispersion test and outlier test are simulation based, using randomized quantile residuals in the DHARMa package
